# Smart Nanostructured Materials for SARS-CoV-2 and Variants Prevention, Biosensing and Vaccination

**DOI:** 10.3390/bios12121129

**Published:** 2022-12-05

**Authors:** Lifeng Wang, Zhiwei Li

**Affiliations:** 1 Suzhou Ninth People’s Hospital, Suzhou Ninth Hospital Affiliated to Soochow University, Suzhou 215000, China; 2 Department of Chemistry, International Institute of Nanotechnology, Northwestern University, Evanston, IL 60208-3113, USA

**Keywords:** SARS-CoV-2, COVID-19 pandemic, smart nanostructures, biosensing, detection, vaccination, immune response

## Abstract

The coronavirus disease 2019 (COVID-19) pandemic, caused by the severe acute respiratory syndrome coronavirus 2 (SARS-CoV-2), has raised great concerns about human health globally. At the current stage, prevention and vaccination are still the most efficient ways to slow down the pandemic and to treat SARS-CoV-2 in various aspects. In this review, we summarize current progress and research activities in developing smart nanostructured materials for COVID-19 prevention, sensing, and vaccination. A few established concepts to prevent the spreading of SARS-CoV-2 and the variants of concerns (VOCs) are firstly reviewed, which emphasizes the importance of smart nanostructures in cutting the virus spreading chains. In the second part, we focus our discussion on the development of stimuli-responsive nanostructures for high-performance biosensing and detection of SARS-CoV-2 and VOCs. The use of nanostructures in developing effective and reliable vaccines for SARS-CoV-2 and VOCs will be introduced in the following section. In the conclusion, we summarize the current research focus on smart nanostructured materials for SARS-CoV-2 treatment. Some existing challenges are also provided, which need continuous efforts in creating smart nanostructured materials for coronavirus biosensing, treatment, and vaccination.

## 1. Introduction

The spread of SARS-CoV-2 has caused the coronavirus 2019 (COVID-19) disease and the pandemic worldwide [1,2,3]. According to the weekly report of the World Health Organization (WHO) as of 25 September 2022, 612 million confirmed cases have been reported, leading to 6.5 million deaths by COVID-19 globally. Although the number of newly reported cases decreases by 11% and new weekly reported deaths decrease by 18% compared with the previous week, there is no convincing evidence and global confidence indicating the end of the COVID-19 pandemic so far [4,5,6]. Additionally, the up-to-date death rate caused by the SARS-CoV-2 is 1.06%, which is higher than the 0.6% in the 1957 influenza pandemic, although the latest rates decreased from case fatality rates of 3.3% about two years ago (as of 9 September 2020). In addition to the coronavirus SARS-CoV-2 itself, there has been a trend of outbreaks of various variants globally, making the current situation unpredictable regarding the spreading of the pandemic [7,8,9,10,11]. It is therefore becoming increasingly important to develop biosensing strategies as well as reliable coronaviral vaccines to prevent and treat the SARS-CoV-2 and its variants of concerns [12].

In many established strategies for coronavirus regulation, nanostructured materials that can actively respond to external stimuli are playing increasingly important roles [13,14,15]. Their unique capabilities to sense and specifically respond to external physical and chemical stimuli represent widely accessible platforms to develop active smart coatings for virus prevention, to be incorporated to advanced sensing devices for coronavirus detection, and to be used for responsive delivery systems for SARS-CoV-2 vaccination [16,17,18]. They have demonstrated their great global success in many aspects in fighting against the COVID-19 pandemic. For example, some emerging techniques have been proposed to functionalize conventional masks with active nanostructures [19]. These innovations are expected to improve prevention efficacy of masks in social activities. Another promising example is the use of photonic crystals and plasmonic nanostructures in developing high-performance virus biosensors [20,21,22]. These responsive nanostructured materials can provide both colorimetric changes for fast, point-of-care detection and spectroscopic readouts for precise quantitative evaluation. In developing coronavirus vaccinations, nanostructured materials have been used to deliver biological species for triggering in vivo immune responses. The introduction of lipid nanostructures as a biocompatible carrier to deliver RNA has been used in commercial vaccines, which have demonstrated the highest efficacy based on clinical data [23,24,25]. Considering these exciting developments, it is critical to summarize the design principles and working mechanisms of this unique set of nanostructured materials in coronavirus regulation. Although there are some reviews providing an overview of materials science in fighting against COVID-19 or summarizing research activities in specific aspects (sensing or vaccination) [26,27,28,29], a focused review on recent advances in creating smart nanostructured materials for the SARS-CoV-2 treatment is necessary to understand the general concepts underlying these remarkable materials and to overcomes existing challenges in tackling COVID-19. 

In this review, we briefly summarize the development of SARS-CoV-2 and the ensuing COVID-19 pandemic in the last two years and overview the progress of smart nanostructured materials in fighting against this widespread virus pandemic from a material science point of view. To understand the current stage of this pandemic and predict its future trends, the genetic information and viral structure will first be discussed. The incurring virus variants and their important genetic mutations will be also introduced, which will be helpful to understand how smart nanostructured materials can be created based on these unique features of the coronavirus. The research activities in developing nanomaterials for virus detection, prevention and vaccination will be discussed in sequence, which emphasizes the vital role of nanostructured materials in preventing and slowing down the existing pandemic. A few important nanostructured materials will be elaborated in each section, including nanostructured membranes for virus filtration, responsive plasmonic nanostructures and photonic crystals for virus detection, and colloidal assemblies and lipid nanoparticles for COVID-19 vaccination. At the end of this review, a perspective on the further development of smart nanostructured materials will be provided in fighting against current COVID-19 pandemic and potential infectious viruses and viral diseases in the future. 

## 2. SARS-CoV-2 and VOCs

**Figure 1:**
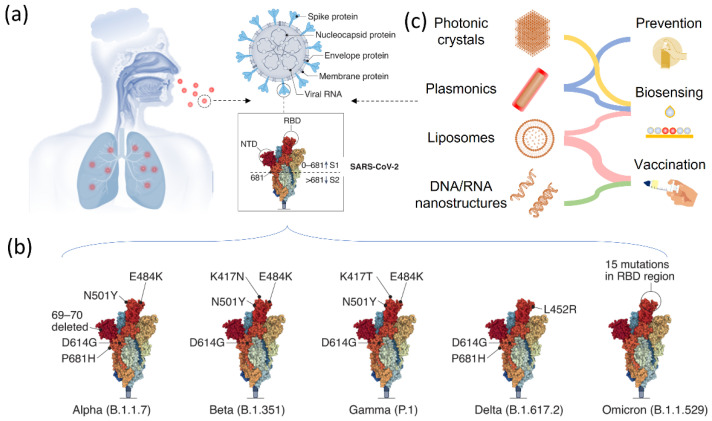
Smart nanostructured materials for SARS-CoV-2 and variants biosensing, treatment, and vaccination. (**a**) Scheme of smart nanostructured materials for COVID-19 treatment. (**b**) The structure of SARS-CoV-2 and the major components, including the S protein, nucleocapsid protein, envelope protein, membrane protein and viral RNA. (**c**) SARS-CoV-2 variants of concerns. Reprinted from [30], with permission from Springer Nature (Berlin/Heidelberg, Germany).

The structure of the RNA virus SARS-CoV-2 is depicted in Figure 1a, with its viral RNA encapsulated in the membrane protein [30,31,32]. It comprises five basic functional structures: a spike protein, envelop protein, membrane protein, nucleocapsid protein, and the viral RNA ranking from the exterior to the interior. More specifically, the SARS-CoV-2 is a positive-strand RNA virus (+ssRNA virus), which contains ~29-kilobase single-stranded, positive-sense genomes made of ribonucleic acid. The spike protein known as S protein on the surface regulates the receptor recognition and cell membrane fusion and therefore is one of the most important functional proteins of the virus [7,33,34]. It has two subunits, S1 and S2 on the virus membrane, with a total number of amino acids larger than 1200. The S1 subunit contains a domain that can recognize and bind to the receptor angiotensin-converting enzyme 2. The S2 subunit is responsible for cell membrane fusion through the formation of a six-helical bundle based on a two-heptad repeat domain [35]. Therefore, the S protein has been extensively studied so far for developing vaccines for tackling the coronavirus pandemic, for investigating immune responses, and for tracking genetic mutations among various variants [36,37,38]. Among these complex structures and diverse amino acid constituents, only a small amino acid stretch is directly related to the interactions between the receptor-binding domain and the enzyme 2 receptor of the host cells. Figure 1b shows the key mutations on the S protein that are noted in all VOC so far, indicating the important role of the S protein in virus mutation and vaccination. 

Compared with other RNA virus, SARS-CoV-2 has a slower mutation rate, with two single-letter mutations per month [39,40]. However, due to the rapid spread and large number of infected patents, a variety of variants have been observed globally as SARS-CoV-2 continues to change the vital genetic codes through genetic mutations and viral combinations when replicating their genomes [41,42]. One remarkable feature of these existing variants is that they have one or more genetic mutations being different from the SARS-CoV-2 and the other variants. If a variant evolves through the combination of genetic codes from two different variants, it can be categorized as a recombinant. Although there are many variants, a lineage can sometimes be recognized, which contains virus variants derived from the same ancestor. According to WHO, more than 4000 variants of SARS-CoV-2 have been reported [43]. The major concern regarding these variants is the escape or hamper of these virus variants from the established immune responses by previous infection or SARS-CoV-2 vaccination [44,45,46]. Based on the genetic modification of the various SARS-CoV-2 variants, they can be divided into three categories to facilitate the necessary attention for the policy determination and efficient treatment, including variants under monitoring, variants of interest (VOI), and variants of concerns (VOC). More specifically, these three types of virus variants are ranked based on the virus genetic changes that are predicted or known to alter a few important properties of the coronavirus, including transmissibility, disease severity, immune responses, and the therapeutic and diagnostic outcomes. Depending on their genetic mutations and the virus spread, it may vary significantly over time.

Understanding the genetic information and biological properties is a prerequisite for developing effective administration strategies and vaccination for SARS-CoV-2 and its variants. To this end, responsive or smart nanostructured materials are playing an important role in different aspects. As shown in Figure 1c, various nanostructured materials, including photonic crystals, plasmonic nanostructures, lipid nanoparticles, and DNA/RNA nanostructures have been studied and used in research and in clinics for the prevention, biosensing, and vaccination of SARS-CoV-2 and its variants. In response to external stimuli or surrounding environmental changes, these materials can show perceivable or detectable signals, which represents an open platform for developing high-performance sensors [47,48,49]. Plasmonic nanostructures and photonic crystals are two representative materials in this regard, which offer programmable optical signals for the sensing and detection of the virus and biomolecules [50,51,52,53,54]. Therefore, they have been extensively used for detecting SARS-CoV-2 and its various variants in current research. One remarkable feature of these nanostructured optical sensors is the readable outputs and colorimetric sensing in response to virus exposure, which significantly facilitates point-of-care and fast detection of the SARS-CoV-2 in a flexible time scale. Moreover, they do not require additional energy input to perform the test, which greatly extends the availability of these biosensors in daily use. Performing quantitative analysis is also possible on these optical sensors by taking advantage of various spectroscopies. Another great success in fighting against the SARS-CoV-2 is the use of nanostructures for virus vaccination, which delivers biological molecules to trigger the immune reactions inside the body. To this end, cationic lipids containing nucleic acids and virus-mimicking nanoparticles for accomplishing S protein delivery are two remarkable examples, demonstrating at the level of fundamental research and clinical trials the great success of nanostructured vaccines in bringing the coronavirus under control, in preventing viral infection, and in reducing disease severity [55]. 

## 3. Nanostructured Materials for COVID-19 Prevention

**Figure 2:**
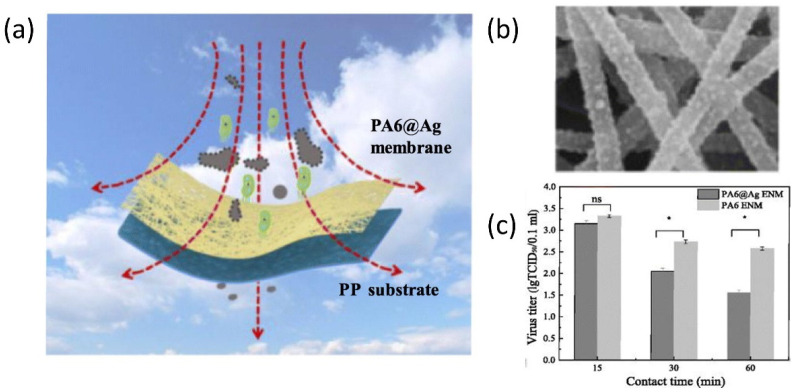
Responsive nanostructured materials for potential COVID-19 treatment and prevention. (**a**) Schematic illustration of nanostructured filter. (**b**) The SEM image of the nanostructured fibers decorated with Ag nanoparticles. (**c**) The viral titer measurement at different contact time. ns: not significant difference; * *p* < 0.05. Reprinted from [72], with permission from Elsevier Inc (Amsterdam, The Netherlands).

One effective way to slow down virus spread is to physically isolate the infectious viruses that are suspended in air. A few common practices nowadays include keeping social distance and wearing personal protective equipment (masks, gloves, face shields, and protective suits). In addition to these common practices, researchers are seeking ways to prevent the spread of the coronavirus using nanostructured filters or coatings, which aim to reduce the number of virus particles suspended in air by capturing them on demand. In the classic design of air filters, filters with regular pores allow selective transport to particulate matters of different sizes. Only particulate matters or nanoscale particles with sizes smaller than the pore diameters can pass through the filters, leaving larger ones blocked and separated. This working mechanism is operational for particulate matters as well as biological species. Functionalizing the filtering materials represents an advanced technique to improve the efficiency [56,57,58]. For example, the top-down fiber manufacturing is a typical method to prepare functional filters, which can be explained by a Brownian diffusion mechanism [59,60,61]. An Al-coated conductive fibrous filter demonstrated an efficiency of >99.99% nanoparticle capture by using electrostatic interactions [62]. However, these strategies require the additional integration of a nanogenerator set and some filters also need ultra-high voltage, which limits their practical use. To overcome these existing challenges, a self-powered filter based on ionic liquid polymer composites was developed with improved hydrophilicity and conductivity, high absolute electrostatic potential, and power generation ability to remove nanoparticles and particulate matters [63]. This self-powered filter was prepared by polymerizing a hydrophilic copolymer on a melamine-formaldehyde (MF) resin sponge. Such highly porous structures allow polluted air to flow through the filter without too much pressure drop while enhancing the particle and virus-removal efficiency, owning to their high surface areas and porosity. This filter demonstrates a high efficiency in removing particulate matters by generating a strong electric field under a low voltage of 3 V. Such a low voltage could be supplied by a silicon solar panel, granting this filter great potential in creating self-powered wearable cleaning devices. Functionalizing nanofibers with active nanostructures will provide additional antibacterial and antiviral properties in addition to passive filtration [64,65,66,67]. To this end, Ag nanoparticles have been long recognized for their excellent antibacterial performances [68,69,70,71]. A typical scheme for classic air filter with antibacterial and antiviral properties is shown in Figure 2a [72]. In this work, a polar polymer, PA6, was made into nanofibers by electrospinning and deposited on a polypropylene substrate. Ag nanoparticles were decorated on the fibrous membranes through a impregnation method (SEM in Figure 2b). Such nanostructured films were used as active filters, which removed suspended bacteria and virus based on the antibacterial and antiviral properties of the guest Ag nanoparticles (Figure 2c). 

**Figure 3:**
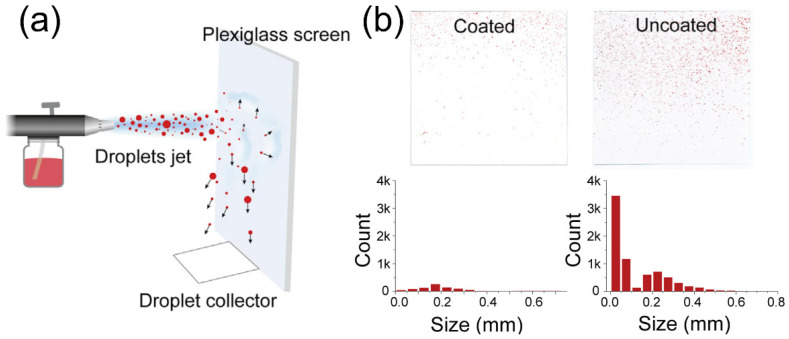
Nanostructured coating for COVID-19 treatment and prevention. (**a**) Schematic illustration of spray experiment for accessing the treatment performance. (**b**) Photos and corresponding histograms showing the droplet marks and their size distributions, respectively. Reprinted from [75], with permission from Elsevier Inc.

Another technique that has been established to remove virus or particles is to actively capture these pollutants in the air with smart coatings [73,74]. The purpose of this design is to remove pathogen-laden respiratory droplets released from nearby patients, which is expected to slow down the spread of pathogens and reduce the transmission of coronavirus. To this end, a cosmetic ingredient-based formulation has been reported to form conformal coatings on surfaces of different materials, compositions, shapes, roughness, and wettability, which can enhance the aerosol-capturing capability [75]. This work introduced polyelectrolytes as coating materials that can increase the wettability by the droplets, delay the elastic recovery of deformed droplets for enhanced deposition, and absorb water quickly from the captured droplets to avoid releasing. To demonstrate these effects, an analytical model was built, which used air-spray to mimic droplet formation (Figure 3a). This quantitative modal demonstrates an enhanced efficiency for droplet-capturing, since the coated surfaces had a lower count of droplets of different sizes (Figure 3b).

## 4. Responsive Nanostructured Materials for Viral Disease Biosensing 

Since responsive nanostructured materials can exhibit optical or electronic signals in response to external stimuli, they have been extensively exploited in developing biosensors for coronavirus detection. Based on the physical properties of the nanostructures, different nanosensing platforms have been established, including colorimetric sensing, fluorescent sensing, SERS sensing, electrochemical sensing, and piezoelectric sensing. The first three optical biosensing platforms provide measurable optical signals while the last two sensing platforms produce electric signals for detecting coronavirus. Table 1 summarizes existing techniques that have been used for COVID-19 detection and diagnosis, which demonstrates the great success of responsive nanostructured materials in developing point-of-care and high-performance biosensors for SARS-CoV-2 detection. As conventional diagnosis methods, CT scanning, X-ray imaging, and magnetic resonance imaging (MRI) have been used for the early diagnosis of COVID-19 in the current pandemic. They are noninvasive and provide three-dimensional (3D) scanning and imaging to identify infections potentially caused by the SARS-CoV-2. However, these methods are non-specific to the sequence of the coronavirus and therefore cannot identify the types of the variants. Moreover, these techniques use expensive facilities that can only be operated by trained technicians [51,76,77]. The reverse transcription-polymerase chain reaction (RT-PCR) and the clustered regularly interspaced short palindromic repeats (CRISPR) are two advanced techniques for highly sensitive and accurate, sequence-specific detection of the coronavirus. However, they are time-consuming, costly, and can only be performed by trained personnel. In the past two years, many biosensors based on nanostructures have been developed to overcome these challenges of conventional diagnosis methods and proved for clinical use and point-of-care detection of the coronavirus. These nanostructures provide colorimetric signals or sensitive electric signals for detecting the virus. The diverse surface chemistry used in these sensing platforms also allows sequence-specific screening and identification of the virus types of the variants. 

To illustrate the working mechanism and surface chemistry of responsive nanostructures used in different biosensing systems, we summarize representative nanostructures in SARS-CoV-2 biosensors in Table 2. Depending on the physical properties of nanomaterials, responsive nanostructures can be developed into different sensing platforms, including colorimetric biosensors, fluorescent biosensors, and electrochemical biosensors. Au nanoparticles have been widely used in colorimetric biosensors because of their LSPR effect and optical properties. Generally, the involved bioconjugation mechanism for functionalizing molecules to sense and recognize biomolecules is thiol chemistry and carbodiimide chemistry. Many biomolecules can be crosslinked to the Au nanoparticle surface through the Au-S or Au-N bonds to target the S proteins, antibodies, or specific DNA sequences. Many Au nanoparticle-based lateral flow immunoassays have been issued with emergency use authorization for COVID-19 diagnosis [78]. These test kits provide fast (within a few minutes), highly sensitive detection of the coronavirus in point-of-care biosensing. In addition, quantum dots and upconversion nanoparticles have been used in fluorescent biosensors for detecting antibodies, proteins of coronavirus due to their strong emission, narrow emission peaks, and tunable wavelengths. Carbon nanostructures have good conductivity and can be used for fabricating electrochemical biosensors. They provide highly sensitive coronavirus detection with low detection limit. For both carbon nanostructures, quantum dots, and upconversion nanoparticles, the carbodiimide crosslinking chemistry is the most used method to bond target molecules for coronavirus biomolecules recognition. 

**Figure 4:**
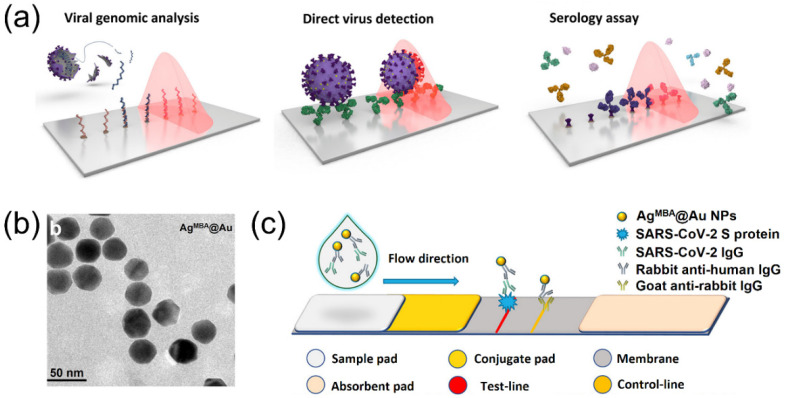
Responsive photonic crystals and plasmonic nanostructures for COVID-19 biosensing. (**a**) Schematic illustration of the virus biosensing and detection. Reprinted from [94], with permission from the American Chemical Society. (**b**) TEM image of the Ag@Au nanoparticles for biosensing of SARS-CoV-2 IgG. (**c**) Schematic illustration of detection principle of a test strip. Reprinted from [105], with permission from American Chemical Society.

Generally, three strategies have been developed to detect respiratory virus using different biosensing and detection platforms (Figure 4a) [94]. The first and most well-established strategy is to detect the genome of the virus through nucleic acid amplification tests (NAATs), which mainly includes polymerase chain reaction (PCR) and its many derivatives [95,96,97]. Direct detection of the intact virus or fragments is the second method in modern virus biosensing, which is mostly realized through the recognition of viral antigens (structural proteins of the virus). The third approach to detecting the existing virus is to target specific antibodies that are produced by the infected hosts after virus infection, which is famous as serological testing. Compared with traditional virus culture methods, these current methods have higher sensitivity and produce accessible data that is easy to analyze [98,99]. For example, the PCR technique can amplify and detect specific sequences of the coronavirus nucleic acids, providing easily accessible diagnosis and remarkably high efficiency. In fighting against the coronavirus in the current COVID-19 pandemic, fast access to the specific sequence of the virus enables the design and commercialization of highly specific PCR kits for point-of-care biosensing and quantitative virus load analysis. Antigen-targeted biosensing uses pre-designed recognition elements, particularly specific antibodies that are reactive to the target antigens, to capture and recognize exposed proteins in the virus antigens. Two famous examples in this regard are the S protein in the coronavirus and the hemagglutinin glycoprotein in the influenza virus [100,101]. Early techniques include immunochromatography (IC) and flow assays, which provide enough sensitivity for quantitative analysis [102,103,104]. Some derived techniques have been commercialized for individual test kits or point-of-care diagnosis of respiratory viruses. 

### 4.1. Responsive Plasmonic Nanostructures for Biosensing of Coronavirus

Plasmonic nanostructures are well-known for their unique optical properties [106,107,108]. Under light irradiation, the free electrons in the plasmonic nanostructures, particularly nanoparticles of Au, Ag and Cu, form resonant oscillation with the electric field of the light, inducing remarkable localized surface plasmon resonance (LSPR) [109]. The resonant frequency and absorption peak position are determined by nanoparticle size, morphology, and chemical components [110,111,112]. A variety of synthetic methods are available now to prepare plasmonic nanostructures, with their LSPR peak positions tunable from visible to near infrared regions. Additionally, assembling plasmonic nanoparticles of different shapes into superstructures is another important approach to actively tuning the LSPR strength and peak position [53]. In a few carefully prepared systems, plasmonic assemblies were used as plasmonic rulers to detect nanoscale distance changes with extremely high spatial resolution [113,114]. For anisotropic nanostructures, like nanorods and nanodiscs, their LSPR is responsive to their orientation. Taking nanorods for example, depending on the relative orientation of the nanorods to the polarization of the light, their LSPR has longitudinal and transverse modes [115,116]. These interesting optical properties have been used for developing high-performance mechanochromic and thermochromic sensors [116,117] or smart imaging contrast agents in photoacoustic imaging and optical coherence imaging [118,119]. The resonant frequency of plasmonic nanostructures is also highly susceptible to surrounding environments. For example, any changes in the surrounding refractive index or dielectric constant will induce peak shift and sometimes colorimetric responses, which has been extensively exploited in colorimetric sensors for detecting biological molecules and infectious viruses [120]. If plasmonic nanostructures are coupled with other optical probes, e.g., fluorescent molecules or Raman molecules, they may greatly enhance the optical signal and improve the signal-to-noise ratio of these probes through different chemical processes and physical mechanisms [121,122,123]. For example, surface-enhanced Raman spectroscopy (SERS) uses plasmonic nanostructures to enhance the Raman scattering of nearby molecules, which has been broadly used in the detection and biosensing of biological molecules and various viruses [124,125,126,127,128,129].

Plasmonic Au and Ag nanostructures have been used in biosensors due to their excellent optical properties and widely tunable LSPR positions from visible to near-infrared regions [130,131,132]. Compared with Au nanostructures, Ag nanomaterials have stronger LSPR and therefore are more favorable in developing high-performance biosensors [133,134,135,136]. However, Ag nanostructures are not stable compared to materials made of Au because Ag are susceptible to oxidizers and can be easily oxidized to cations at ambient conditions. To overcome these limitations, a few strategies have been developed in colloidal synthesis, including forming alloy or core/shell nanostructures with Au [137]. One remarkable feature of these composite nanostructures is that the nanostructures exhibit strong LSPR of Ag while maintaining good chemical and thermal stability. For example, ultrathin Au nanoshells were coated on Ag nanoparticles such that the shell can protect the Ag core from being etched without compromising its plasmonic performance (Figure 4b) [105,138]. These nanoparticles were used as probes to detect immunoglobulin G (IgG) antibodies of the SARS-CoV-2. To specifically recognize the coronavirus, these core/shell plasmonic nanoparticles were firstly modified with anti-human antibodies, which was captured by the S protein on the coronavirus T line. These biosensing probes were then integrated to lateral flow immunoassay (as shown in Figure 4c) for colorimetric detection of the virus, providing qualitative and quick examination of the presence of target coronavirus. In addition, quantitative analysis is also possible on this platform by performing SERS measurement with a low detection limit (0.22 pg/mL) [105].

**Figure 5:**
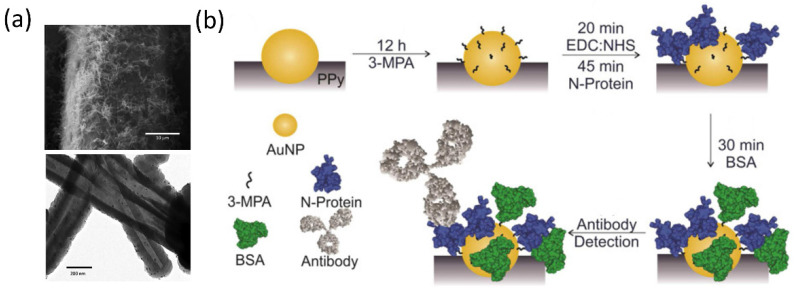
Responsive plasmonic nanostructures for biosensing. (**a**) SEM (top panel) and TEM (bottom panel) of the polypyrrole nanotubes decorated with Au nanoparticles. (**b**) Schematic illustration of the preparation and working principle of the biosensors for antibody detection. Reprinted from [141], with permission from Elsevier Ltd.

Developing electrochemical sensors for biosensing is also possible by introducing plasmonic nanoparticles as sensing platforms, which has been extensively used in impedimetric biosensors [139]. This technique uses electrochemical impedance spectroscopy to sufficiently recognize and quantitatively analyze target biomolecules [140]. One remarkable feature of these nanostructures in electrical biosensing is their much larger surface areas compared with bulk materials, which is expected to improve the electrochemical activities and sensitivity of the systems. In this regard, combining plasmonic nanostructures with conductive polymers is a practical approach to designing electrical biosensors. For example, small Au nanoparticles decorated on polypyrrole nanotubes have been developed as a biosensing platform to detect anti-SARS-CoV-2 nucleocapsid protein monoclonal antibodies (Figure 5a) [141]. This was realized by modifying the surface of the conductive nanotubes with SARS-CoV-2 nucleocapsid protein through covalent bonds (Figure 5b). The impedimetric detection performance of this biosensor demonstrates a detection limit of 0.4 ng/mL of the monoclonal antibody.

**Figure 6:**
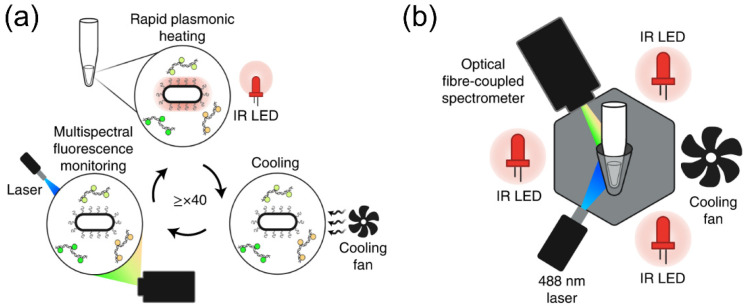
(**a**) Schematic of multiplexed real-time plasmonic reverse-transcriptase polymerase chain reaction. The Au nanorods are suspended in solution in a PCR tube, which can rapidly absorb light from the light source and convert it to heat, allowing for fast PCR thermal cycling. (**b**) Schematic illustration of the instrument. A PCR tube is surrounded by low-cost optical components. The main components of the instrument include a thin-walled PCR tube surrounded by three IR LED modules, a cooling fan, and a 488 nm laser and spectrophotometer setup for fluorescence detection. Reprinted from [131], with permission from Springer Nature.

PCR as an advanced nucleic acid amplification technique has been broadly used in biosensing of virus genome [142,143,144]. One great advantage over other sensing platforms is the high sensitivity and extremely low detection limit thanks to the ability of amplifying target nucleic acids in solution reactions [145,146,147,148]. It also offers real-time detection of target molecules, fast sensing of virus genomes, and precise quantitative analysis of the infected units. However, current techniques require complicated setups for precise temperature control and nucleic acid amplification, which hinders its broad use in point-of-care diagnostics. To overcome this challenge and develop PCR miniaturization techniques, plasmonic Au nanoparticles have been recently introduced to the PCR systems [131], which are used as heating agents in the reaction by taking advantage of their excellent photothermal conversion under light irradiation [149,150,151,152,153]. The working mechanism of this real-time plasmonic PCR is shown in Figure 6a. The plasmonic thermocycling is realized by infrared excitation of the Au nanoparticles for rapid heating in a reaction vessel containing PCR chemistry, fluorescent probes, and the plasmonic nanoparticles. A 12 V fan is then used to cool the reaction. To detect the fluorescent signals in real-time, a 488 nm laser is used as a portable light source and an optical fiber-coupled spectrometer is used as integrated parts to measure the optical signals (Figure 6b). This modified PCR system allows rapid detection of the RNA of SARS-CoV-2 in human saliva and nasal specimens with 100% sensitivity and 100% specificity.

### 4.2. Responsive Photonic Crystals for Biosensing of Viral Disease

Photonic crystals are periodic superstructures that can exhibit structural colors at a particular wavelength [138,154,155]. Although they can exhibit tunable colors, the coloration mechanism is based on diffraction of light at particular wavelength, which is different from plasmonic absorption and scattering of metallic nanostructures. The bottom-up colloidal assembly and top-down lithography are two general methods to create photonic crystals [156]. The periodic arrangement of materials with different refractive indexes creates photonic bandgap, which diffract light at this stopband. The fundamental physical principle to understand this optical effect is Bragg’s law, 2ndsinϴ = kλ [156,157]. In this equation, n and d are the effective refractive index and periodicity of the photonic crystals, respectively, and ϴ is the incident angle in the measurement. The k and λ are diffraction order and wavelength of diffracted light. Based on this simple equation, it can be predicted that the structural color and diffracted light wavelength are determined by the physical properties of the photonic crystals and surroundings, which can be used to design responsive photonic crystals for detecting virus and biological species [158,159,160,161,162,163,164]. Two of the most used strategies in this regard include the changes of surrounding dielectrics and control over the periodicity of the photonic crystals [165].

**Figure 7:**
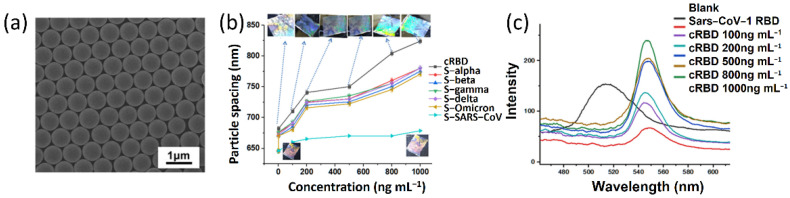
Responsive photonic crystals for biosensing. (**a**) SEM image of two-dimensional polystyrene array. Reprinted from [167], with permission from American Chemical Society. (**b**) Particle spacing measurements with the Debye diffraction ring diameter obtained with a laser pointer. (**c**) Measurements of the cRBD from the APC-sensor using a UV−vis spectrometer. Reprinted from [166], with permission from American Chemical Society.

One recent work used polystyrene nanospheres as building blocks and assembled them into photonic crystals [166]. The hexagonal packing of the polystyrene nanospheres is shown in Figure 7a, which can be commonly observed in the close-packing of nanospheres [167]. These photonic crystals can be embedded into functional hydrogels that are modified by functional groups or molecules for biosensing. Polyacrylamide hydrogel was used in this work to form a continuum matrix, in which the polystyrene photonic crystals were incorporated inside. Afterwards, single-stranded DNA aptamers were modified to the hydrogel matrix, which can selectively bind to the consensus receptor-binding domain of the S protein of the SARS-CoV-2 virus and the variants in saliva samples. Such specific binding induces swelling of the hydrogel and leads to the increase in the periodicity of the photonic crystals and the redshift of their structural colors (Figure 7b,c). This biosensing platform is rapid and convenient, providing both perceivable colorimetric changes and spectroscopic detection of the SARS-CoV-2.

**Figure 8:**
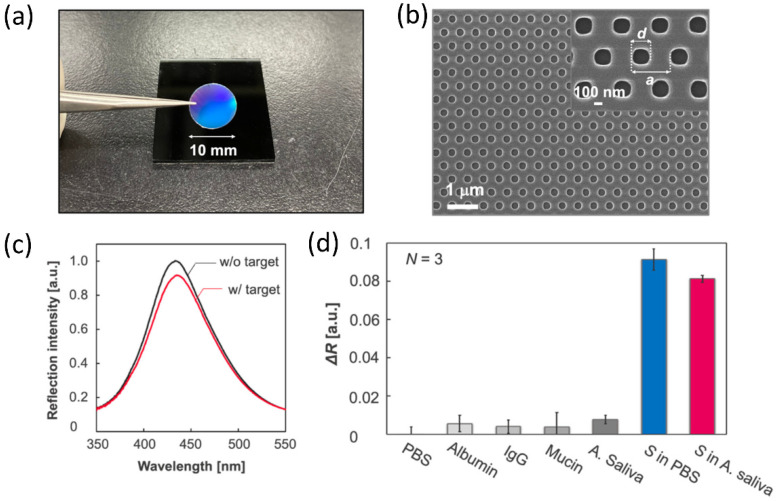
Responsive photonic crystals for label-free specific detection of SARS-CoV-2 spike proteins. (**a**) Image of the photonic crystal chip. The diameter of the chip used for the sensor was 10 mm. (**b**) SEM image of the photonic crystal surface. (**c**) Reflection spectrum of the photonic crystal before and after incubation in a sample solution containing spike proteins. (**d**) Detection selectivity for the spike proteins. Reprinted from [169], with permission from MDPI.

In addition to colloidal self-assembly, the top-down lithography is another well-established method to prepare photonic crystals with desirable colors [168]. This method can produce two-dimensional photonic crystals on solid substrates in a large scale. One advantage of photonic crystals made by lithography over those prepared by colloidal self-assembly is their ease in being incorporated into functional devices. The easy handling of the photonic films and many accessible post-treatment methods enable these photonic structures to be broadly used in developing sensing photonic chips. They can provide both colorimetric responses for fast screening of analytes and spectroscopic detection for precise content measurement. For example, a polymer-based imprinted photonic crystal was developed recently for simple and fast optical detection and quantification of the S protein of the SARS-CoV-2 [169]. Using a nanoimprint technique, the photonic film can be made at a centimeter scale (Figure 8a). The brilliant blue colors demonstrate the perfect order of the nanostructures on the surface of the photonic film. It contains 230 nm hole arrays arranged in a hexagonal phase with a lattice constant of 460 nm (Figure 8b). Then, an anti-SARS-CoV-2 antibody was modified on the surface of the photonic films for selective virus detection. The working mechanism of this photonic chip is based on the decrease of diffraction peak intensity after specific attachment of the coronavirus to the surface of the photonic films. By carefully analyzing such a decrease (Figure 8c), it is possible to evaluate the detection limit, selectivity of this sensing photonic chips and to estimate the content of the coronavirus. By incubating these types of photonic chips with a chosen culture medium, it is possible to verify the selectivity and response to different contaminants. As shown in Figure 8d, this photonic chip has no response to inorganic chemicals in the phosphate buffer solution while having weak response to contaminant proteins largely because of the nonspecific absorption of these large biological molecules to the film surface. However, photonic chips incubated with S protein have much higher changes in reflection intensity, which demonstrates the good selectivity of these photonic chips in sensing SARS-CoV-2.

**Figure 9:**
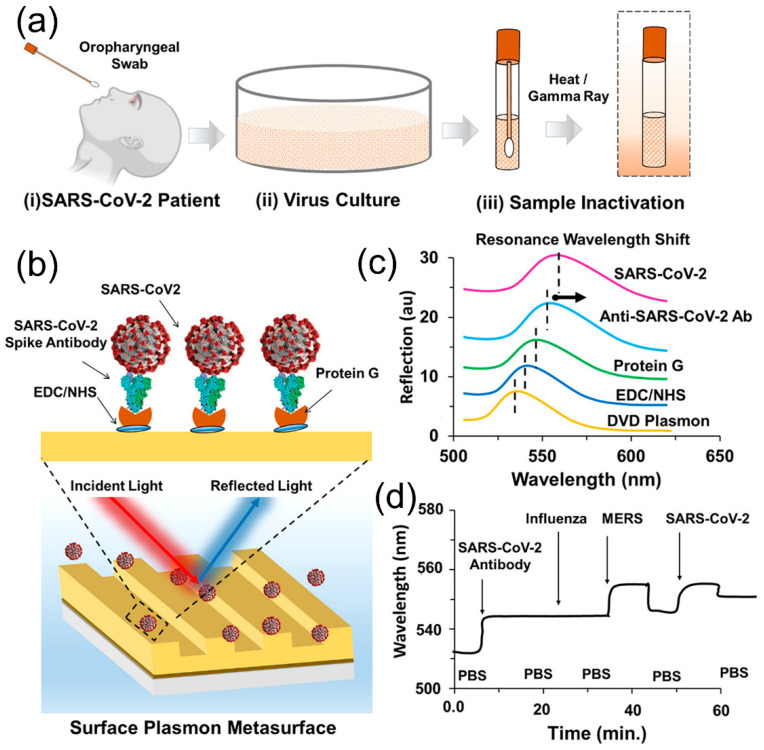
Plasmonic metasurface sensor based on periodic structures for SARS-CoV-2 detection and biosensing. (**a**) Schematic illustration of SARS-CoV-2 sample collection, virus culture, and heat or gamma irradiation steps for inactivated virus preparation. (**b**) Schematic illustration showing the surface plasmon resonance-based SARS-CoV-2 detection. (**c**,**d**) Optical response of the proposed sensor is based on the resonance wavelength shift based on molecular binding that allows differential identification of SARS and influenza from their specific and nonspecific binding events. Reprinted from [179], with permission from American Chemical Society.

By coupling periodic structures with plasmonic excitation, it is possible to offer new optical responses in biosensing [170,171,172]. Lattice plasmon resonance and Fano resonance are two representative examples, which provide highly sensitive platforms to detect biological species [173,174,175,176,177,178]. One reliable way to manufacture periodic plasmonic nanostructures is to deposit thin layer of metals on the surface of a template featuring periodic structures. Such metasurfaces, once coupled with spectrometers, are able to provide specific and sensitive optical signals in response to bonding to target biological molecules, e.g., proteins, DNA, antibodies, or viruses. A recent study introduces a simple strategy to prepare a cost-effective, large-scale biosensing platform for SARS-CoV-2 detection [179]. In its design, commercial blank DVD disks were used as starting templates for manufacturing the plasmonic platform. Any plastic or polymer layers on the surface of the commercial CDC were removed, leaving the plastic-templated metasurface with plasmonic metal-coated surface grating exposed for further functionalization. Such metasurfaces were further assembled into a microfluidic chip through attachment of tubing and adhesive layers for biosensing SARS-CoV-2. To realize specific targeting of the coronavirus antibody, protein and virus particles, a layer-by-layer functionalization was introduced to link SARS-CoV-2 antibody to the metasurface. To test the biosensing performance of the plasmonic chips, a standard procedure was established to deactivate the coronavirus through heat and gamma irradiation (Figure 9a). Since the metasurface was modified by the SARS-CoV-2 spike antibody, it can specifically recognize and quantify the SARS-CoV-2 particles (Figure 9b). When broadband light is incident on the plasmonic chip metasurface, the optical resonance can be directly observed and monitored in the reflected light in real-time. The reflection peak position due to the resonance response redshifts during the layer-by-layer surface modification and after specific bonding to the coronavirus and virus-related biological species (Figure 9c). Such an optical response can be carefully monitored and collaborated for the fast detection of interactions and binding of biomolecules and quantification of target molecule concentration (Figure 9d). This simple design allows for the quantitative detection of antibodies, proteins, or the whole virus with high sensitivity and specificity. In addition, this work can efficiently distinguish the SARS-CoV-2 from other similar RNA virus such as influenza, representing a highly accessible biosensing platform for the real-time detection of SARS-CoV-2 and pathogens.

## 5. Nanotechnology in Viral Disease Vaccination

**Figure 10:**
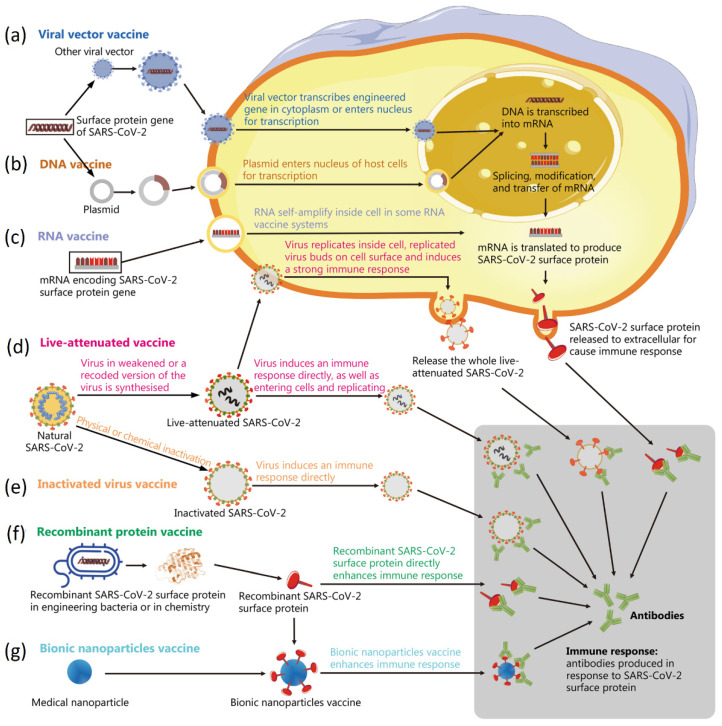
Schematic diagram showing the principles of various vaccines: (**a**) viral vector vaccine, (**b**) DNA vaccine, (**c**) RNA vaccine, (**d**) live-attenuated vaccine, (**e**) inactivated virus vaccine, (**f**) recombinant protein vaccine, and (**g**) bionic nanoparticles vaccine. Reprinted from [182], with permission from Elsevier.

At the current stage, vaccination is still the most effective technology to regulate the COVID-19 pandemic [180,181]. It can activate the immune responses inside the body such that the possibility for infection can be reduced even when exposed to SARS-CoV-2. There are three types of COVID-19 vaccines that have been approved for clinic use in the United States, which include message RNA (mRNA), viral vector and protein subunits. These vaccines are injected in human bodies to trigger immune responses and to recognize the virus that causes COVID-19. In addition to these three types of vaccines used in clinic, researchers have developed many different vaccines. These vaccines in various formats induce immune responses in different physiological pathways (Figure 10) [182]. Specifically, vaccines that enter cells for triggering immune responses include viral vector vaccine, DNA vaccine, RNA vaccine, and live-attenuated vaccine. For inactivated virus vaccine and recombinant protein vaccine, they do not need to enter cells for training immune reactions. In addition, the viral vector vaccines are created by incorporating SARS-CoV-2 antigen species into viruses that have low pathogenicity (Figure 10a) [183,184,185]. DNA vaccines use plasmid as a vector to enter the nucleus of the host cells for transcription (Figure 10b) [186,187,188]. In developing mRNA vaccines, the S protein gene of the SARS-CoV-2 will be encoded into the mRNA, which is encapsulated into biocompatible nanoparticles (e.g., lipid nanoparticles). These antigens are produced in vitro and delivered into the human cells and then translated into a protein antigen by the cells to train in vivo immune response (Figure 10c) [189,190,191]. Weakening or completely inactivating the virus while retaining their surface proteins is a direct way to produce immune responses inside bodies (Figure 10d,e) [192,193,194]. In other strategies, the S protein on the surface of SARS-CoV-2 is delivered through engineered bacteria, assembled nanostructures (Figure 10f) or medical nanoparticles (Figure 10g) to directly enhance immune responses [33,195,196,197].

**Figure 11:**
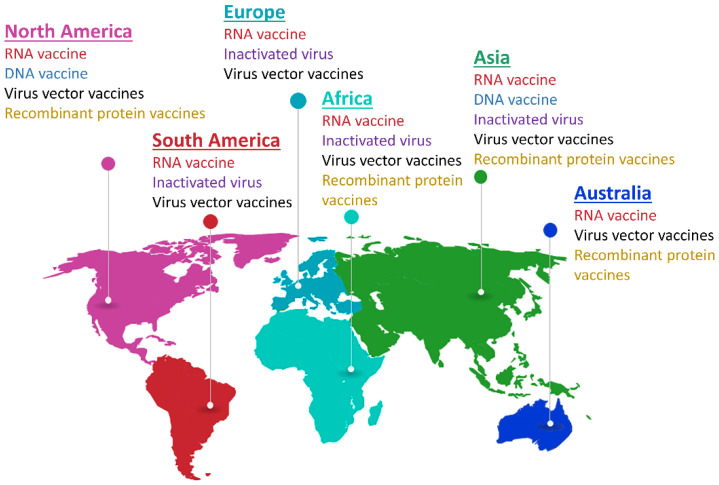
Global COVID-19 vaccines map. The use and development of COVID-19 vaccines is summarized in different continents.

**Figure 12:**
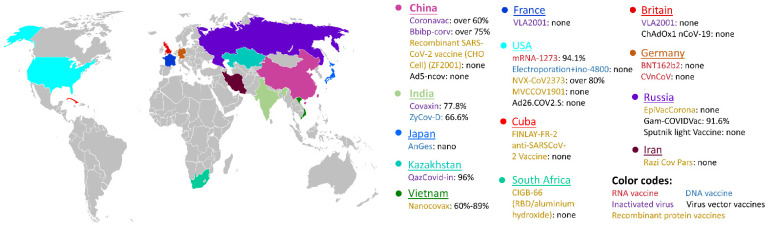
Global vaccine efficacy maps in different countries. The vaccines that have been used in different countries are color-coded by the vaccine types (RNA vaccine, DNA vaccine, inactivated vaccine, virus vector vaccine, and recombinant protein vaccine). In each highlighted country, the name and efficacy of the vaccines are provided in this map.

These vaccines have been investigated worldwide during the COVID-19 pandemic. Figure 11 depicts the global vaccine map, which summarizes the vaccination strategies for different continents. It can be found that the RNA vaccines and virus vector vaccines are the two mostly exploited vaccines for SARS-CoV-2 protection. Moreover, in this global vaccination map, both conventional inactivated vaccines and emerging RNA vaccines have been approved for clinical use in different countries. One great effort in vaccine research is to evaluate the vaccine efficacy in different phases and administration strategies, which is normally difficult to conduct in reality. Indeed, the statistics on vaccine efficacy are affected by many factors, including the phase of vaccine development, statistical method, patient nationality, gender, age, etc. However, the access to global and local vaccine efficacy is critically important to evaluate the effectiveness of the vaccines, to determine further administration strategies, and to develop modified vaccines for coronavirus variants. In the last two years, many reports have been provided globally regarding vaccine effectiveness, enabling a summary of the effectiveness and reliability of different vaccine techniques. Figure 12 provides a global overview of vaccine efficacy of the five different vaccine types, including inactivated vaccines, RNA vaccines, DNA vaccines, virus vector vaccines, and recombinant vaccines (or protein subunit vaccines). In Asian countries, such as China and India, the efficacy of conventional inactivated vaccines is between 60% and 80%. The mRNA vaccines have demonstrated an efficacy at ~94% in USA, which are among the most effective vaccines in the clinic. Moreover, NVX-CoV2373 is proven to be one of the recombinant protein vaccines and its efficacy is evaluated to be over 80%. The detailed information of a few representative vaccines is summarized in Table 3. Most vaccines still need low-temperature environments in transportation and storage, which sets a limitation for their wide implementation, particularly for remote regions and some underdeveloped countries. Therefore, designing vaccines that work at ambient temperatures is critical to further popularize the vaccination rate globally, which is one research goal for chemists and biologists.

### 5.1. Delivery of mRNA Using Lipid Nanoparticles

**Figure 13:**
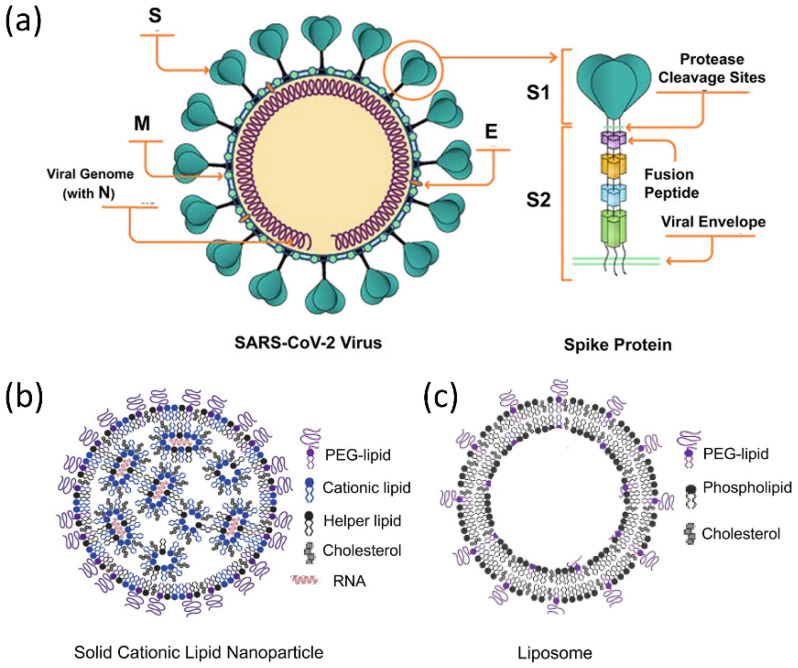
Responsive nanostructured materials for COVID-19 treatment and prevention. (**a**) Schematic illustration of the SARS-CoV-2 components for generating protective antiviral immune responses. (**b**) The proposed structure of LNP–siRNA formulations containing ionizable amino-lipids within inverted micellar structures surrounding siRNA. (**c**) Liposomal formulations contain an aqueous core with electron densities consistent with the exterior of the liposome. Reprinted from [55], with permission from American Chemical Society.

The mRNA vaccines use lipid nanoparticles to deliver mRNA that can trigger the production of S proteins in the bodies [198]. Two commercial mRNA vaccines in this regard are from Pfizer-BioNTech and Moderna. The mRNA is genetically engineered in a scientific lab and then encapsulated in the lipid nanoparticles, which trains cells to express the S proteins found on the surface of the SARS-CoV-2 (Figure 13a,b). Notably, the lipid nanoparticles are different from classic liposome nanoparticles that have a lipid bilayer and a liquid core (Figure 13c) [199]. In the formation of lipid nanoparticles, the cationic lipids will complex with nucleic acids, producing electron-dense cores inside the lipid nanoparticles. To encapsulate and deliver mRNA, the lipid nanoparticles have a few typical components, which include an ionizable aminolipid having electrostatic interactions with RNA and are responsible for the hydrophobic inverted micelles formation, cholesterol to promote close packing of each component, helper lipids for stabilizing cell membranes, and lastly the PEG-lipid serving as surface hydrating layer to enhance particle stability. Compared with neutral liposomes in delivering oligonucleotides, the charged lipids and their strong electrostatic interactions with oligonucleotides significantly increase the loading and delivery efficiency.

In clinical practice, these mRNA vaccines are injected into upper arm muscle or upper thigh depending on the age of the vaccinated patients. After the mRNA enters the muscle cells, it will use the machinery of the cells to generate pieces of the S proteins, which will be expressed on the cell surface. The mRNA itself will be broken down into pieces and be removed by the bodies. Due to the expression of the S protein on the surface of normal cells, it will trigger immune responses inside the body and the immune system recognizing the foreign proteins will produce antibodies and activate immune cells to fight infections.

### 5.2. Assembly of Viral Protein Subunits for Vaccination

**Figure 14:**
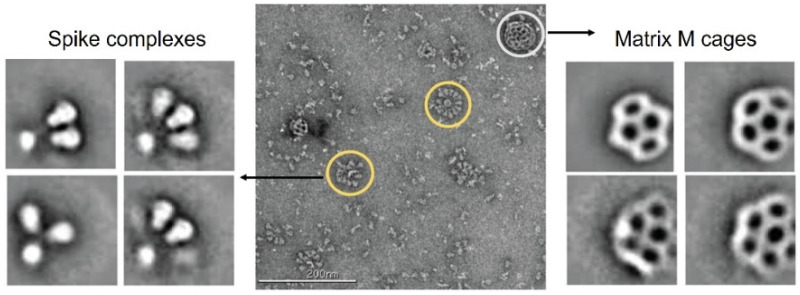
COVID-19 vaccine development through self-assembly of stabilized, full-length SARS-CoV-2 S protein subunits into nanoparticles. Negative stain electron microscopy of the full-length spike (reconstituted in PS 80) admixed with the cage-like Matrix-M component (from plant origin). The spike rosettes are circled in yellow, and Matrix-M adjuvant cages are circled in white. Reprinted from [206], with permission from American Association for the Advancement of Science.

Based on the structures of the SARS-CoV-2, the S glycoprotein is the focus and target for developing coronavirus vaccines because it is expressed on the virus surface and therefor is the main target of the host immune defense systems [200,201,202]. In addition to the delivery of mRNA using lipid nanoparticles, developing viral S protein subunits is another approach to coronavirus vaccination [55]. In this regard, Novavax has created a S protein subunits vaccine, which entered phase 3 clinical trials in 2021 [203]. After one year of development, the Novavax COVID-19 vaccine (NVX-C0V2373) has been approved for clinical use [204,205]. Additionally, the Novavax COVID-19 Omicron vaccine that is designed for the most widespread variant of the SARS-CoV-2 is in phase 3 clinical trials. The NVX-C0V2373 vaccine is a nanoparticle vaccine self-assembled and derived from the recombinant expression of full-length S proteins, with their structures shown in Figure 14 [206]. Under the presence of Sorbitol 80, the free 2-P full-length S protein assemble into prefusion Spike complexes, from dimer of trimers, trimer of trimers to large oligomers of trimers. Previous studies have pointed out that the tighter clusters made of the protein subunits improve the immunogenicity [207,208]. The Novavax COVID-19 vaccine (NVX-C0V2373) also has a proprietary adjuvant, Matrix M cages (right panel in Figure 14), which is made of 40 nm honeycomb-like nanoparticles. These nanoparticles are derived from plant saponins and further mixed with cholesterol and a phospholipid. An important feature of the Novavax protein subunit vaccines is the transportability and above-freezing storage temperature. Therefore, the relative high storage temperature compared with the mRNA vaccines make them more accessible and easier to be transported globally.

## 6. Summary

In summary, we review recent progress and research activities in developing smart or responsive nanostructured materials for SARS-CoV-2 prevention, biosensing, and vaccination. Extensive research has demonstrated that this unique set of materials plays important roles in different aspects in fighting against the global COVID-19 pandemic. In the first part of this review, we briefly introduced the structures, function, and properties of SARS-CoV-2, which is a prerequisite to design smart nanostructures on demand and understand their working mechanism in pandemic regulation. The use of nanotechnology in collection and filtration is overviewed, which can remove the suspended coronavirus from public environment and therefore reduce the potential of infection when exposed to nearby virus carriers. In the next section, several advanced biosensing platforms were presented, which include responsive plasmonic nanostructures and photonic crystals for highly efficient detection of virus. In the last part, two well-established vaccinations were deliberated, while considering the importance of nanomaterials in developing stable and efficient vaccines for SARS-CoV-2 and its many variants.

Despite these exciting developments in fighting against COVID-19, there are still many limitations and challenges in the virus prevention, biosensing, and vaccination. In coronavirus prevention, developing commercial masks with reusable capability or virus-killing functions is an existing challenge considering the large consumption of disposable masks during the current pandemic. In addition to physical absorption and blocking of the coronavirus, these emerging masks are expected to actively kill the SARS-CoV-2 through some established methods, like photothermal killing or the photodynamic effect [18,209,210]. Solving this challenge can reduce the resource consumption, eliminate environmental pollution caused by the great pandemic, and most importantly increase protection efficacy of masks. Moreover, current common practices in fighting against the SARS-CoV-2 include wearing masks and keeping social distance. It remains challenging to develop active coating materials that can reduce environmental contamination and capture suspended coronavirus in the air. For coronavirus biosensing, improving the sensitivity and specificity and simplifying current sensing systems for point-of-care detection need to be solved in the future. Introducing stimuli-responsive nanomaterials is a promising way to improve the detection limits of conventional biosensing techniques. One good example in this regard is the use of plasmonic nanoparticles for SERS-based viral and tumor detection. In terms of developing commercial miniaturized test devices, some existing consumer products are good examples to develop similar techniques for coronavirus detection, which are particularly important in the current pandemic situation where anti-corona drugs are not available for reliable COVID-19 treatment. For instance, smart watches are commercially available for measuring body temperature, blood oxygen level, and heart beat rate, which greatly facilitates general healthcare. Small portable devices can also be used for detecting glucose levels in diabetes treatment and monitoring chemicals in body sweat. Developing similar devices for instant and low-cost diagnostics of COVID-19 is expected to provide powerful tools for both personal daily testing and COVID-19 pandemic regulation. In designing coronavirus vaccines, the vaccination for the mutation of the SARS-CoV-2 and various variants is still limited by the unpredictable biological structures of the variants and the lack of a generalized vaccine for treating different variants, although current vaccines have demonstrated convincing efficiency in activating immune responses for SARS-CoV-2. At the current stage, neutralizing SARS-CoV-2 by monoclonal antibodies can block infection and provide effective therapeutic protection for COVID-19. However, a recent study reports that several authorized monoclonal antibodies have reduced neutralizing potency towards variants in bodies, particularly the Omicron variant [211]. Therefore, current monoclonal antibody design limits its use in fighting against various variants of concerns. A potential approach to solve this challenge is cocktails of two or more target monoclonal antibodies for multiple sites of vulnerability on the S protein. The simultaneous delivery of the multiple antibodies targeting different epitopes can recognize variants of different mutations and therefore provide cross-protection against existing and potential variants [212,213].

Developing new sensing platforms and integrating existing biosensing devices are still necessary for daily detection and point-of-care diagnostics. To this end, designing wearable sensing devices based on electrochemical or colorimetric sensors is a promising approach to the coronavirus detection. In addition, the continuous mutation of the SARS-CoV-2 calls for additional efforts in designing generalized vaccines that can be used for triggering the body’s immune reactions for different variants. These continuous efforts in diverse research communities will greatly benefit the prevention, biosensing, and vaccination of SARS-CoV-2 and variants and future disease treatments.

## Figures and Tables

**Table 1:** Comparison between the conventional detection techniques and the biosensing techniques based on responsive nanostructures.

Diagnostic Techniques		Advantages	Disadvantages
Conventional diagnostic techniques	CT scan	Early screening of infection, no sampling, non-invasive	Non-specific, X-ray exposure, operational only by technicians
	X-ray imaging	Low cost, no sampling, non-invasive	Non-specific, false negatives, operational only by technicians, X-ray exposure
	MRI	Non-invasive infection monitoring, 3D imaging	Costly, only available in technical labs
	RT-PCR	High accuracy and sensitivity, sequence-specific sensing of coronavirus	Long detection time, high cost, only operational for trained experts
	CRISPR	Lost cost, highly sensitive, integrated to portable devices	Necessary specific CRISPR sequences
Biosensing techniques based on responsive nanostructures	LSPR sensing	Colorimetric changes, easy operation, low cost, available for point-of-care detection, fast detection	Large-scale production of noble metal nanoparticles, complicated fabrication
	SERS sensing	Highly sensitive, specific to virus, quantitative detection	Raman spectroscope needed
	Fluorescent biosensing	Highly sensitive and accuracy, low detection limit	Possible fluorescence quenching
	Electrochemical biosensing	Highly sensitive, label-free	Energy consumption, possible incorrect positives, low reproducibility
	Piezoelectric biosensing	Highly sensitive and specific, label-free and fast detection	Complicated sample preparation and pretreatment

**Table 2:** Summary of responsive nanostructures in biosensing of the SARS-CoV-2.

Type	Nanostructures	Surface Chemistry	Target	Sensitivity	Specificity	References
Colorimetricbiosensors	Au nanoislands	Thiol chemistry	DNA sequences of SARS-CoV-2	1.32 × 10^5^ copies/μL	–	[79].
	Au nanoparticles	Thiol chemistry	N-gene	1 copy µL^−1^	–	[80].
	Au thin films	Carbodiimide chemistry	Anti-SARS-CoV-2 antibodies	1 μg/mL	–	[81].
	Au nanoparticles	–	S and nucleocapsid protein	96.7%	100%	[82].
	Magnetic beads/Au nanoparticles	Au−N and Au−S bonds and hydrophobic interactions	N protein	69 fg mL^−1^	–	[83].
	Au nanoneedles array	Thiol chemistry	Virus via S protein	80 copies mL^−1^	–	[84].
	Cellulose nanobeads	–	Nucleocapsidprotein	88.4%	100%	[85].
	Au nanoparticles	–	S protein	100%	97.5%	[86].
Fluorescentbiosensors	Lanthanide-Doped Nanoparticles	Carbodiimide chemistry	Anti-SARS-CoV-2 IgG	–	–	[87].
	SiO_2_@QDs	Carbodiimide chemistry	SARS-CoV-2antigen	5 pg/mL	–	[88].
	CdSe/ZnS quantum dots	Carbodiimide chemistry	Antibodies	90%	100%	[89].
Electrochemical biosensors	Au@Fe_3_O_4_/carbon electrodes	Thiol chemistry	Viral RNA	3 aM	–	[90].
	GO-Au NS	Carbodiimide chemistry	Glycoproteins	0.0048 μAμg.mL^−1^.cm^−2^	–	[91].
	Graphene-ssDNA-AuNP/Au Electrode	Thiol chemistry	Viral RNA	231 (copies/μL)^−1^	~100%	[92].
	rGO/3D printed 3D electrode	Carbodiimide chemistry	Antibodies to spike S1 protein	1 × 10^−12^ M	–	[93].

**Table 3:** Summary of vaccine information (efficacy, name or manufacturer, development phase, efficacy, dose, and storage condition) for different types of vaccines worldwide. Note that RT means room temperature.

Type	Company or Vaccine Name	Phase 3	Efficacy (%)	Dose	Storage (°C)
mRNA vaccine	Sinovac	NCT04582344	50	2 (14−day interval)	2–8
	Pfizer/BioNTech	NCT04368728	95	2 (21 days apart)	−70
	Moderna	NCT04470427	94	2 (28 days apart)	−20
	CureVac	NCT04652102	47	2 (28 days apart)	2–8
DNA vaccine	AnGes	NCT04655625	none	2 (14− and 28−day interval)	RT
Inactivated virus	Sinopharm	NCT04510207	79	2 (21−day interval)	2–8
Virus vector vaccines	AstraZeneca	NCT04324606	62–90	2 (28−day interval)	2–8
	Gameleya	NCT04530396	91.6	2 (21−day interval)	−18
	Johnson & Johnson	NCT04505722	66–85.4	1	2–8
Recombinant protein vaccines	Novavax	NCT04636697	60–89	2 (21−day interval)	2–8

## Data Availability

Not applicable.
